# Increased Number of Tc17 and Correlation with Th17 Cells in Patients with Immune Thrombocytopenia

**DOI:** 10.1371/journal.pone.0026522

**Published:** 2011-10-24

**Authors:** Yu Hu, Dao-xin Ma, Ning-ning Shan, Yuan-yuan Zhu, Xin-guang Liu, Lei Zhang, Shuang Yu, Chun-yan Ji, Ming Hou

**Affiliations:** 1 Department of Hematology, Qilu Hospital, Shandong University, Jinan, People's Republic of China; 2 Department of Hematology, Provincial Hospital Affiliated to Shandong University, Jinan, People's Republic of China; 3 Department of Orthopedics, Qilu Hospital, Shandong University, Jinan, People's Republic of China; Centre de Recherche Public de la Santé (CRP-Santé), Luxembourg

## Abstract

**Background:**

IL-17-secreting CD8+ T cells (Tc17 subset) have recently been defined as a subpopulation of effector T cells implicated in the pathogenesis of autoimmune diseases. The role of Tc17 and correlation with Th17 cells in the pathophysiology of immune thrombocytopenia (ITP) remain unsettled.

**Design and Methods:**

We studied 47 ITP patients (20 newly-diagnosed and 27 with complete response) and 34 healthy controls. IL-17-producing CD3+CD8+ cells (Tc17) and IL-17-producing CD3+CD8− cells (Th17) were evaluated by flow cytometry and expressed as a percentage of the total number of CD3+ cells. Specific anti-platelet glycoprotein (GP) GPIIb/IIIa and/or GPIb/IX autoantibodies were measured by modified monoclonal antibody specific immobilization of platelet antigens. Peripheral blood mononuclear cells of ITP patients were isolated, incubated in the presence of 0, 0.25, 0.5, or 1 µmol/L of dexamethasone for 72 h, and collected to detect Tc17 and Th17 cells by flow cytometric analysis.

**Results:**

IL-17 was expressed on CD3+CD8− and CD3+CD8+ T cells. The percentages of Tc17 and Th17 cells in newly-diagnosed patients were significantly elevated compared to controls, and Tc17 was decreased after clinical treatment. The Th17∶Tc17 ratio was significantly lower in newly-diagnosed patients compared with controls, and was increased in patients who had complete response. There was a significantly positive correlation between Tc17 and Th17 cells in the control group, but not in the ITP patients. A positive correlation existed between Tc17 and the CD8∶CD4 ratio, as well as CD8+ cells in patients with ITP. The frequencies of Tc17 were marginally higher in autoantibody-negative patients than autoantibody-positive patients. Moreover, both Tc17 and Th17 cell percentages decreased as the concentration of dexamethasone in the culture media increased in ITP patients.

**Conclusions:**

Tc17 and the Th17 subset are involved in the immunopathology of ITP. Blocking the abnormally increased number of Tc17 may be a reasonable therapeutic strategy for ITP.

## Introduction

Immune thrombocytopenia (ITP) is characterized by a low platelet count, which is the result of increased platelet destruction and insufficient platelet production [1]. The pathophysiology of ITP is heterogeneous and complex. T-lymphocyte abnormalities are considered important in patients with ITP. Many cellular mechanisms of immune modulation have been described in patients with ITP, such as T helper 1 (Th1) bias [2], a decreased number or defective suppressive function of regulatory T cells [3], and platelet destruction by cytotoxic T lymphocytes (CTLs) [4]. More recently, utilizing a new animal model of ITP it has been shown that CTLs mediate thrombocytopenia [5]. However, the mechanism of cellular immune abnormalities in patients with ITP remains unclear.

Th17 cells comprise a novel Th cell subset characterized by the production of IL-17 [6]. Th17 cells have been shown to play a crucial role in the induction of autoimmune diseases, including rheumatoid arthritis, experimental autoimmune encephalomyelitis (EAE), and allergen-specific responses [7]. In our previous work, we determined the number of Th17 cells in the peripheral blood of ITP patients and healthy controls by flow cytometry through intracellular cytokine analysis, and demonstrated for the first time that Th17 cells are elevated in ITP patients [8]–[10]. More recently, a completely new subset of IL-17-secreting CD8+ effector cells, so-called Tc17, have been described with a quite different phenotype from other cytolytic T cells. Similar to Th17 cells, naïve CD8+ T cells differentiate into Tc17 cells in the presence of IL-6 and IL-21 along with TGF-β [7]. Tc17 cells secrete IL-17A and IL-17F, and little or no IFN-r and IL-4. In contrast to Tc1 or Tc2, Tc17 express neither granzyme B nor perforin, and have no cytolytic activity against antigen (Ag)-loaded targets [11].

Tc17 have been shown to be involved in various conditions. Tc17 can protect mice against lethal influenza challenge, while Tc17 protection is accompanied by greater neutrophil influx into the lung than in Tc1-injected mice [11]. Tc17 have also been shown to play an important role in controlling the growth of B16 malignant melanoma in mice via the recruitment of neutrophils [12]. Moreover, Tc17 promote acute graft-versus-host disease (aGVHD) in bone marrow transplantation, and the occurrence of aGVHD is reduced through decreased IL-17 secretion by T cells [13]. Tc17 are also implicated in the pathogenesis of some human autoimmune diseases, such as psoriasis [14] and systemic lupus erythematosus (SLE) [15]. However, the characteristics of Tc17 and the correlation between Tc17 and Th17 in ITP patients have not been systematically investigated.

To further investigate the possible role of Tc17 and Th17 cells in the pathogenesis of ITP, we measured the number of Tc17 and correlated the number to Th17 cells and evaluated the clinical relevance.

## Methods

### Ethics Statement

Our research was approved by the Medical Ethical Committee of Qilu Hospital of Shandong University. An informed consent document was obtained from each participant. The informed consent stated that the unused portion or excess of the peripheral blood drawn from patients for complete blood cell counts was as the source of Tc17 in ITP research, or the peripheral blood drawn from healthy subjects for this research was voluntary.

### Patients and Controls

Forty-seven ITP patients (25 females and 22 males; age range, 16–80 years; median age, 41 years), including 20 newly-diagnosed and 27 ITP patients who had complete response (CR) after glucocorticoid therapy, intravenous gamma globulin, and/or splenectomy were enrolled in this study. Enrollment took place between May 2010 and June 2011 in the Department of Hematology of Qilu Hospital (Jinan, China). The platelet counts ranged between 1 and 70×10^9^/L, with a median count of 24.57×10^9^/L. The megakaryocyte counts in the bone marrow smear (1.5 cm×3.0 cm) ranged between 26 and 560, with a median count of 173. Patients with diabetes, hypertension, cardiovascular diseases, pregnancy, active or chronic infections, or connective tissue diseases, such as SLE, were excluded. All of the patients met the diagnostic criteria of ITP, as previously described [16]. Thirty-four healthy controls were included (18 females and 16 males; age range, 20–58 years; median, 32 years). The platelet counts of healthy controls ranged from 132 to 308×10^9^/L, with a median count of 229×10^9^/L.

### Flow Cytometric Analysis

Intracellular cytokines were studied by flow cytometry to identify the cytokine-producing cells. Briefly, heparinized peripheral whole blood (400 µl) with an equal volume of Roswell Park Memorial Institute (RPMI)-1640 medium was incubated for 4 h at 37°C in 5% CO_2_ in the presence of 25 ng/mL of phorbol myristate acetate (PMA), 1 µg/mL of ionomycin, and 1.7 µg/mL of monensin (all from Alexis Biochemicals, San Diego, CA, USA). PMA and ionomycin are pharmacologic T cell-activating agents that mimic signals generated by the T cell receptor (TCR) complex and have the advantage of stimulating T cells of any Ag specificity. Monensin was used to block intracellular transport mechanisms, thereby leading to an accumulation of cytokines in the cells.

After incubation, the cells were stained with PE-Cy5-conjugated anti-CD3 and fluorescein isothiocyanate-conjugated anti-CD8 monoclonal antibodies at room temperature in the dark for 15 min to delimit CD4+ T cells because CD4 was down-modulated when cells were activated by PMA. After surface staining, the cells were stained with PE-conjugated anti-IL17 monoclonal antibodies after fixation and permeabilization according to the manufacturer's instructions. Isotype controls were given to enable correct compensation and confirm antibody specificity. Stained cells were analyzed by flow cytometric analysis using a FAC Scan cytometer equipped with CellQuest software (BD Bioscience PharMingen, San Jose, CA, USA).

### Addition of dexamethasone (DEX) to peripheral blood mononuclear cells (PBMCs) from patients with ITP

PBMCs of ITP patients were isolated from heparinized blood by gradient centrifugation (400×g for 20 min) on Ficoll-Paque (Pharmacia Diagnostics, Uppsala, Sweden), and washed twice. Then, PBMCs were adjusted to 1×10^6^/ml in RPMI-1640 culture medium supplemented with 10% heat-inactivated fetal bovine serum at a density of 2×10^6^ cells/well in a 6-well culture plate and incubated in humidified air in 5% CO_2_ at 37°C in the presence of 0, 0.25, 0.5, or 1 µmol/L of DEX. After 72 h, the PBMCs were collected to enumerate Tc17 and Th17 by flow cytometric analysis.

### Anti-platelet autoantibody determination

All plasma samples were stored at −20°C prior to use. The specific anti-platelet GPIIb/IIIa and/or GPIb/IX autoantibodies were analyzed by modified monoclonal antibody-specific immobilization of platelet antigens (MAIPA), which was carried out as previously described in detail by Hou et al. [17].

### Statistical Analysis

The results are expressed as the median (range). Comparisons between the two groups were assessed by the Wilcoxon rank-sum test, and Spearman's test was used for correlation analysis. All tests were performed by SPSS (version 16.0; SPSS, Inc., Chicago, IL, USA). *P* value<0.05 was considered statistically significant.

## Results

### Elevated Tc17 and Th17 cells in ITP patients

**Figure 1:**
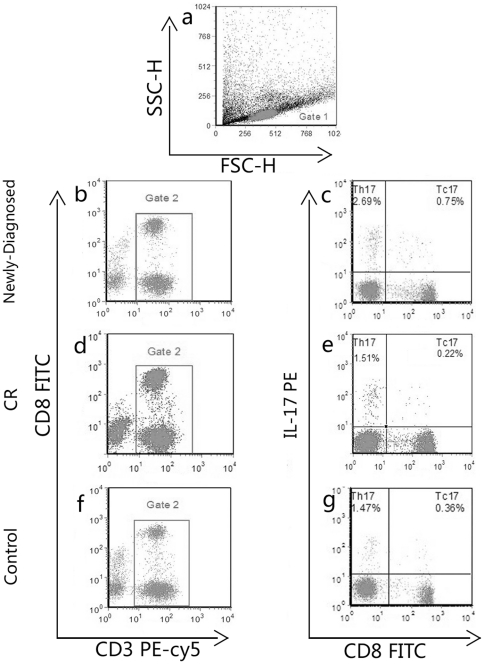
The percentages of circulating Tc17 and Th17 cells in ITP patients and controls. Heparinized peripheral whole blood from all subjects was stimulated with phorbol myristate acetate, ionomycin, and monensin for 4 h, then stained with labeled antibodies as described in the Design and Methods section. (a) Lymphocytes were gated by flow cytometry in Gate 1. (b,d,f) CD3+ T subsets were gated by flow cytometry; the plots in intern box Gate 2 represent CD3+ T cells. (c, e, g) The percentages of circulating Tc17 and Th17 cells from ITP patients and controls; the percentage of positive cells is shown in each panel.

**Figure 2:**
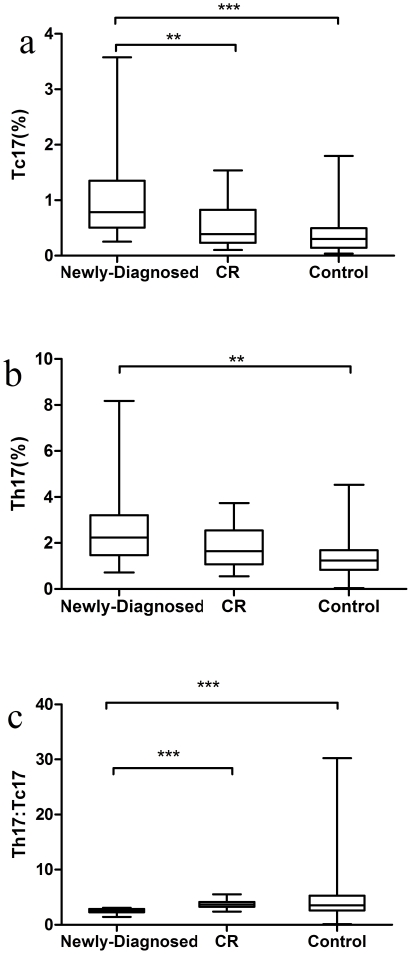
Tc17 and Th17 cells, and the Th17∶Tc17 ratio in ITP patients and controls. (a) Significantly elevated percentage of Tc17 was found in newly-diagnosed ITP patients (median, 0.79%; range, 0.25–3.58%) compared to controls (median, 0.30%; range, 0.04–1.80%; ***P<0.0001) and CR patients (median, 0.39%; range, 0.10–1.54%; **P = 0.0017). (b) Statistically elevated percentage of Th17 cells was found in newly-diagnosed ITP patients (median, 2.24%; range, 0.72–8.18%) compared to controls (median, 1.19%; range, 0.04–4.53%; **P = 0.0016; [similar to our previous study]). (c) The Th17∶Tc17 ratio in newly-diagnosed patients (median, 2.5850; range, 1.4221–3.0714) was 0.76-fold lower than controls (median, 3.3852; range, 1.0000–5.8571; ***P<0.0001) and 0.70-fold lower than CR cases (median, 3.6771; range, 2.3896–5.5000; ***P<0.0001).

We analyzed the frequencies of Tc17 and Th17 cells based on cytokine patterns after *in vitro* activation by PMA/ionomycin in short-term cultures. The expression of a typical dot plot of IL-17 gated on CD3+ T cells in ITP patients and healthy controls is shown in Fig. 1. IL-17 was expressed on CD3+CD8− and CD3+CD8+ T cells. Compared with healthy controls (median, 0.30%; range, 0.04–1.80%), the percentage of Tc17 was significantly increased in the newly-diagnosed patients (median, 0.79%; range, 0.25–3.58%; *P*<0.0001), while there was no significant change in the patients with CR (median, 0.39%; range, 0.10–1.54%). Tc17 were decreased significantly in patients with CR compared to newly-diagnosed patients (*P* = 0.0017; Fig. 2a). We also demonstrated a statistically significant difference in Th17 between newly-diagnosed patients (median, 2.24%; range, 0.72–8.18%) and healthy controls (median, 1.19%; range, 0.04–4.53%; *P* = 0.0016; Fig. 2b), which was similar to the results we previously reported [9].

We noted a difference in the Th17∶Tc17 ratio among newly-diagnosed patients with CR and the healthy control group. As shown in Fig. 2c, the Th17∶Tc17 ratio in newly-diagnosed patients (median, 2.5850; range, 1.4221–3.0714) was 0.76-fold lower than in the healthy controls (median, 3.3852; range, 1.0000–5.8571; *P*<0.0001) and 0.70-fold lower than in the patients with CR (median, 3.6771; range, 2.3896–5.5000; *P*<0.0001). There were no significant differences in the Th17∶Tc17 ratio in patients with CR compared to healthy controls (*P* = 0.2483).

### Tc17 are positively correlated with Th17 cells in healthy controls, but not in ITP patients

**Figure 3:**
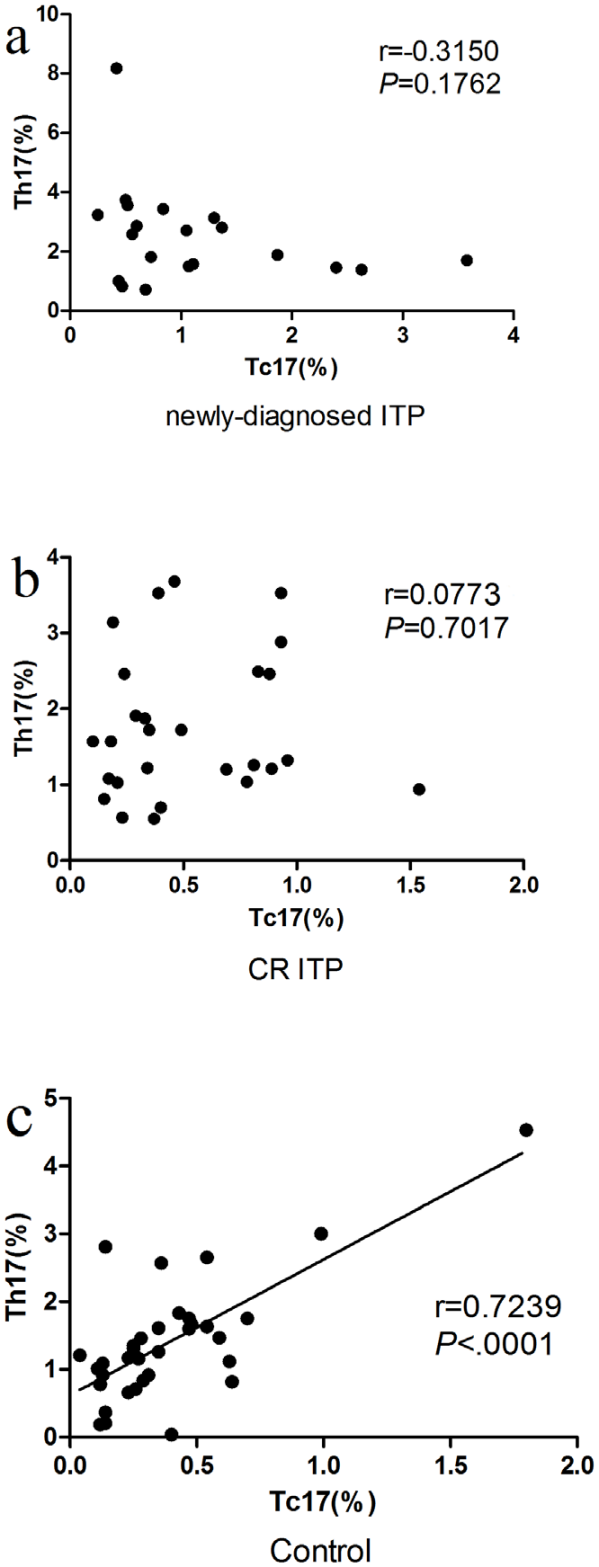
Correlations between Tc17 and Th17 cells in ITP patients and controls. (a, b) No significant correlation was demonstrated in newly-diagnosed (r = −0.3150, P = 0.1762) or CR ITP patients (r = 0.0773, *P* = 0.7017); (c) A positive correlation was shown between Tc17 and Th17 cells in controls (r = 0.7239, *P*<0.0001).

The relationships between Tc17 and Th17 cells in ITP patients and the healthy control group were investigated. As shown in Fig. 3, the frequencies of Tc17 and Th17 cells were positively correlated in healthy controls (r = 0.7239, *P*<0.0001), but not in newly-diagnosed patients (r = −0.3150, *P* = 0.1762) or patients with CR (r = 0.0773, *P* = 0.7017).

### Tc17 are positively correlated with the CD8∶CD4 ratio and the percentage of CD8+ cells in ITP patients

**Figure 4:**
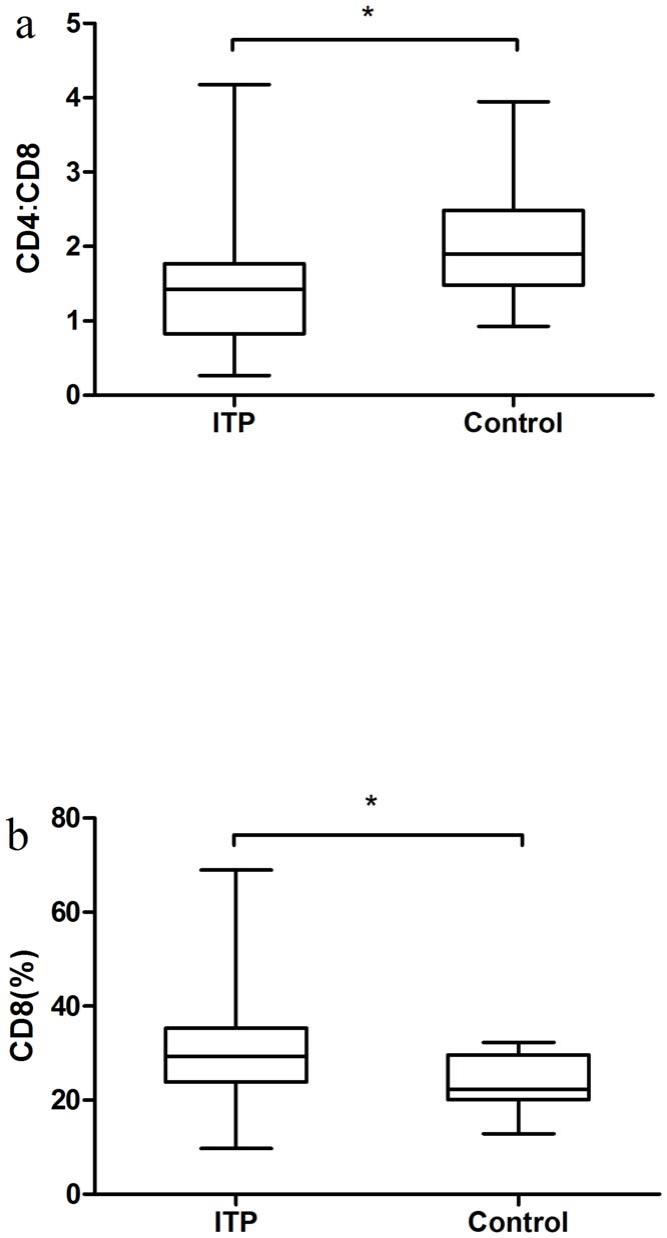
CD8 cells and CD4∶CD8 ratio in ITP patients and controls. (a) Significant difference of the CD4∶CD8 ratios between ITP patients (median, 1.42; range, 0.26–4.18) and control group (median, 1.90; range, 0.93–3.95; *P = 0.029). (b) Elevated percentage of CD8+ T cells in ITP patients (median, 29.34%; range, 9.78%–68.97%) compared with controls (median, 22.35%; range, 12.89%–32.3%; *P = 0.025).

Because the CD4∶CD8 ratio is an important index by which to evaluate immune status, as shown in Figs. 4a and b, we determined the difference in CD4∶CD8 ratios between ITP patients and healthy controls. The ratio of CD4∶CD8 was decreased in ITP patients (median, 1.42; range, 0.26–4.18) compared to healthy controls (median, 1.90; range, 0.93–3.95; *P* = 0.029), and the percentage of CD8+ T cells was increased in ITP patients (median, 29.34%; range, 9.78–68.97%) compared with healthy controls (median, 22.35%; range, 12.89–32.3%; *P* = 0.025).

**Figure 5:**
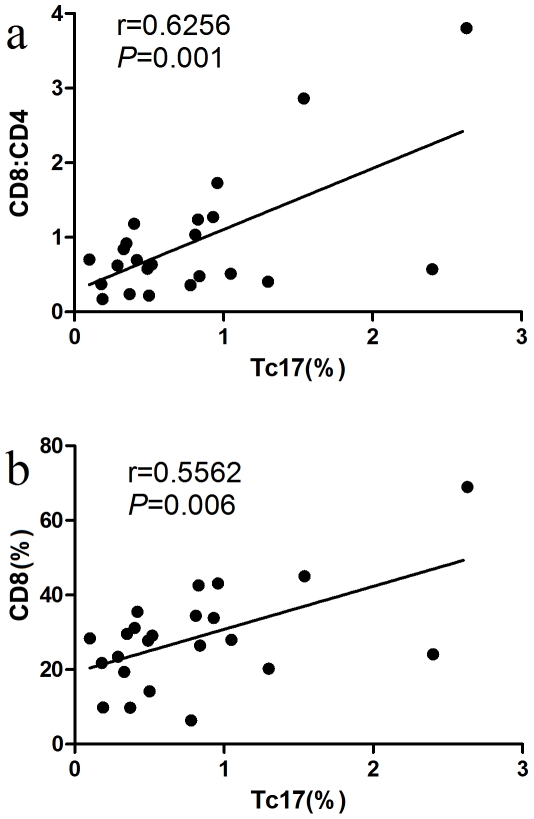
Correlations of Tc17 with CD8 and CD8;CD4 in ITP. Positive correlation of Tc17 with CD8∶CD4 ratio (r = 0.6256, P = 0.001) and positive correlation of Tc17 with percentage of CD8+ T cells (r = 0.5562, *P* = 0.006) in ITP.

In the study we determined the correlation of Tc17 with the CD8∶CD4 ratio, as well as CD8+ cells in ITP patients. As shown in Figs. 5a and 5b, Tc17 and CD8∶CD4 ratios were positively related (r = 0.6256, *P* = 0.001), while Tc17 and CD8+ cells were also positively related in ITP patients (r = 0.5562, *P* = 0.006).

### Marginally elevated Tc17 in autoantibody-negative ITP patients

Circulating anti-platelet autoantibodies are frequently detected in ITP patients. The most common targets of anti-platelet antibodies are the GPIIb/IIIa complex and GPIb/IX. We determined the correlation of anti-platelet GPIIb/IIIa and/or GPIb/IX autoantibodies with Tc17 in ITP patients. The number of Tc17 was marginally elevated in patients who had a negative MAIPA test (median, 1.00; range, 0.18–3.58) compared to patients with a positive MAIPA test (median, 0.50; range, 0.23–0.96; *P* = 0.07).

### Tc17 and Th17 cells are decreased in the presence of DEX in PBMCs of ITP patients *in vitro*

**Figure 6:**
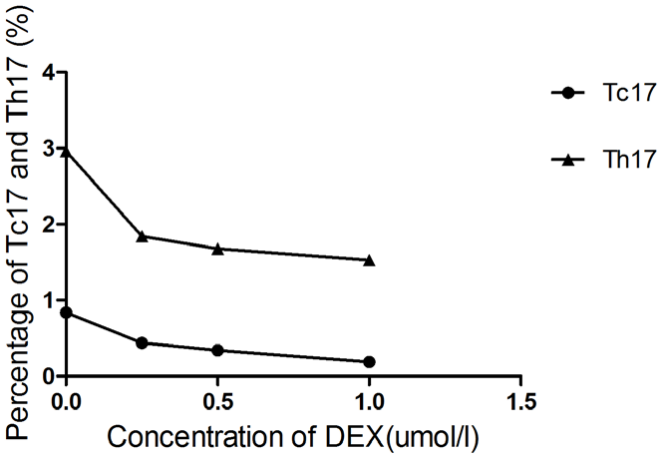
The response of Tc17 and Th17 cells to DEX in ITP patients. The percentages of Tc17 and Th17 cells had a decreasing tendency with the increase in DEX concentration in culture media.

To determine the response of Tc17 and Th17 cells to DEX, we measured the changes in the percentages of Tc17 and Th17 cells from PBMCs of ITP patients in the presence of DEX *in vitro*. As shown in Fig. 6, the percentage of Tc17 and Th17 cells decreased with the increase in the concentration of DEX in culture media.

### Clinical relevance of Tc17 in ITP patients

Because insufficient platelet production by megakaryocytes is a main cause of ITP, we analyzed the association between Tc17 and megakaryocytes or platelet counts in ITP patients. There was no significant correlation between Tc17 and the megakaryocyte count (r = 0.0319, *P* = 0.867). In addition, no significant correlation existed between the number of Tc17 and the platelet count (r = −0.1749, *P* = 0.301).

## Discussion

ITP is an autoimmune disease in which abnormalities in cellular immunity have been clearly demonstrated [18]. Apart from a shift in the Th1/Th2 balance, a new T cell subset (Th17 cells), the primary CD4+ cells producing IL-17, is often regarded as the principal instigator of autoimmune disorders. Th17 cells are increased in patients with chronic inflammatory demyelinating polyradiculoneuropathy (CIDP) [19] and psoriasis [20]. The Tc17 subset is a new unique cell lineage in terms of function and differentiation [21]. There are considerable data showing that Th17 cells are increased in ITP patients [9]. However, only a few studies have focused on Tc17 in autoimmune diseases. More recently, it has been shown that the percentages of Tc17 were increased in psoriatic skin lesions or the peripheral blood of SLE patients, which indicates that this type of CD8+ T cell plays a significant role in the pathogenesis of autoimmune diseases [14],[15]. Tc17 and Th17 cells have also been shown to be infiltrated in positive patch test biopsy samples of patients with allergic contact dermatitis (ACD), indicating that Tc17 and Th17 cells are an integral part in the immunopathology of ACD, regardless of the nature of the triggering allergen [22]. Although Tc17 are aberrant in these autoimmune disorders, it is still not clear whether or not Tc17 are involved in ITP.

To study the role of Tc17 in the development of ITP, Tc17 were examined in the peripheral blood of newly-diagnosed patients, ITP patients who achieved CR, and healthy controls. Our results demonstrated that the number of Tc17 (defined as CD3+CD8+IL-17+) was significantly increased in newly-diagnosed patients, but not in patients who had CR, which indicated the importance of Tc17 in the development of ITP. Moreover, the percentage of Tc17 in ITP patients was decreased with the addition of DEX *in vitro*, strongly suggesting that Tc17 may be involved in the pathogenesis of ITP. Also, the number of Th17 cells was increased in newly-diagnosed ITP patients compared with healthy controls; similar results were produced in our previous research [9]. In the current study we confirmed the increased number of Th17 cells in ITP patients. Moreover, due to the similar trends in increased numbers of Th17 cells and Tc17 in ITP patients, we compared the ratio of Th17∶Tc17 between newly-diagnosed patients, ITP patients with complete response, and healthy controls, and showed that the Th17∶Tc17 ratio was decreased in newly-diagnosed patients compared with healthy controls and patients who had CR. This indicated that Tc17 were increased more predominately than Th17 in patients with active ITP. Thus, we presume that the Tc17 subset might play a more important role in the immunopathology of ITP patients.

In addition, the Tc17 differentiate along a developmental program that is similar to the one described for Th17 cells [23],[24],[25]. The positive correlation of the percentages between Th17 cells and Tc17 in control samples suggests that Tc17 is co-regulated with Th17 cells during differentiation in normal human peripheral blood, and substantially advanced our understanding of T-cell mediated immunity. Th17 cells and Tc17 may play a cooperative or synergistic function under conditions of T cell-mediated immunity; however, no significant correlation existed between Tc17 and Th17 cells in the peripheral blood of ITP patients. These results strongly support the hypothesis that there is a pathologic differentiation of T cell subsets in patients with ITP, which ultimately causes a derangement between Tc17 and Th17 cells.

The CD4∶CD8 ratio is an important index in immune-related diseases [26]. A low CD4∶CD8 ratio may be a sign of immune dysfunction [27]. Interestingly, in the current study we showed that the CD4∶CD8 ratio and percentage of CD8 cells were elevated in patients with ITP compared with healthy controls. Also, Tc17 was positively correlated with the percentage of CD8+ cells and the CD8∶CD4 ratio. The results in the control group were dissimilar. The mechanism underlying the correlation between Tc17 and the CD4∶CD8 ratio or the percentage of CD8 cells should be clarified in future studies.

Platelet-reactive antibodies are not detected in all patients with ITP. A subset of patients do not respond to pharmacologic therapy, antibody-mediated platelet clearance, or B-cell suppression, suggesting the possible involvement of other pathogenic mechanisms, such as Ag shedding, and T cell-mediated platelet destruction or marrow suppression [4]. We detected Tc17 and specific anti-platelet GPIIb/IIIa and/or GPIb/IX autoantibodies in 23 patients by MAIPA. The results showed that the number of Tc17 and titers of autoantibodies had a marginal negative correlation, suggesting cellular and humoral immunity might be complementary in the pathogenesis of ITP.

Taken together, our study demonstrated that the predominant Tc17 might differentiate and develop under the influence of a particular microenvironment and eventually cause an imbalance in Tc17 and Th17 cell subsets in patients with ITP. The Tc17 subset could be decreased with effective clinical treatment. T-cell function derangement has been demonstrated in patients with ITP with abnormal T cells secreting IL-17. The elevated Tc17 may play a potential role in the development and progression of ITP, and blocking the abnormally increased number of Tc17 cells may lead to a novel therapeutic strategy for ITP.

## References

[1] Stasi R, Evangelista ML, Stipa E, Buccisano F, Venditti A (2008). Idiopathic thrombocytopenic purpura: current concepts in pathophysiology and management.. Thromb Haemost.

[2] Semple JW, Milev Y, Cosgrave D, Mody M, Hornstein A (1996). Differences in serum cytokine levels in acute and chronic autoimmune thrombocytopenic purpura: relationship to platelet phenotype and antiplatelet T-cell reactivity.. Blood.

[3] Sakakura M, Wada H, Tawara I, Nobori T, Sugiyama T (2007). Reduced Cd4+Cd25+ T cells in patients with idiopathic thrombocytopenic purpura.. Thromb Res.

[4] Olsson B, Andersson PO, Jernås M, Jacobsson S, Carlsson B (2003). T-cell-mediated cytotoxicity toward platelets in chronic idiopathic thrombocytopenic purpura.. Nat Med.

[5] Chow L, Aslam R, Speck ER, Kim M, Cridland N (2010). A murine model of severe immune thrombocytopenia is induced by antibody- and CD8− T cell–mediated responses that are differentially sensitive to therapy.. Blood.

[6] Harrington LE, Hatton RD, Mangan PR, Turner H, Murphy TL (2005). Interleukin 17-producing CD4(+) effector T cells develop via a lineage distinct from the T helper type 1 and 2 lineages.. Nat Immunol.

[7] Huber M, Heink S, Grothe H, Guralnik A, Reinhard K (2009). A Th17-like developmental process leads to CD8+ Tc17 cells with reduced cytotoxic activity.. Eur J Immunol.

[8] Ma DX, Zhu XJ, Zhao P, Zhao CH, Li XF (2008). Profile of Th17 cytokines (IL-17, TGF-beta, IL-6) and Th1 cytokine (IFN-gamma) in patients with immune thrombocytopenic purpura.. Ann Hematol.

[9] Zhang JB, Ma DX, Zhu XJ, Qu X, Ji CY (2009). Elevated profile of Th17, Th1 and Tc1 cells in patients with immune thrombocytopenic purpura.. Haematologica.

[10] Zhu XJ, Ma DX, Zhang JB, Peng J, Qu X (2010). Elevated interleukin-21 correlated to Th17 and Th1 cells in patients with immune thrombocytopenia.. J Clin Immunol.

[11] Hamada H, Garcia-Hernandez MdeL, Reome JB, Misra SK, Strutt TM (2009). Tc17, a unique subset of CD8 T cells that can protect against lethal influenza challenge.. J Immunol.

[12] Garcia-Hernandez MdeL, Hamada H, Reome JB, Misra SK, Tighe MP (2010). Adoptive transfer of tumor-specific Tc17 effector T cells controls the growth of B16 melanoma in mice.. J Immunol.

[13] Zhao XY, Xu LL, Lu SY, Huang XJ (2011). IL-17-producing T cells contribute to acute graft-versus-host disease in patients undergoing unmanipulated blood and marrow transplantation.. Eur J Immunol.

[14] Res PCM, Piskin G, Boer OJde, van der Loos CM, Teeling P (2010). Overrepresentation of IL-17A and IL-22 producing CD8 T cells in lesional skin suggests their involvement in the pathogenesis of psoriasis.. PLoS ONE.

[15] Henriques A, Inês L, Couto M, Pedreiro S, Santos C (2010). Paiva, Frequency and functional activity of Th17, Tc17 and other T-cell subsets in systemic lupus erythematosus.. Cell Immunol.

[16] Rodeghiero F, Stasi R, Gernsheimer T, Michel M, Provan D (2009). Standardization of terminology, definitions and outcome criteria in immune thrombocytopenia of adults and children: report from an international working group.. Blood.

[17] Hou M, Peng J, Shi Y, Zhang C, Qin P (2003). Mycophenolate mofetil (MMF) for the treatment of steroid-resistant idiopathic thrombocytopenic purpura.. Eur J Haematol.

[18] Semple JW (2002). Immune pathophysiology of autoimmune thrombocytopenic purpura.. Blood Rev.

[19] Chi LJ, Xu WH, Zhang ZW, Huang HT, Zhang LM (2010). Distribution of Th17 cells and Th1 cells in peripheral blood and cerebrospinal fluid in chronic inflammatory demyelinating polyradiculoneuropathy.. J Peripher Nerv Syst.

[20] Kagami SJ, Rizzo HL, Lee JJ, Koguchi Y, Blauvelt A (2010). Circulating Th17, Th22, and Th1 cells are increased in psoriasis.. J Invest Dermatol.

[21] Kondo T, Takata H, Matsuki F, Takiguchi M (2009). Cutting edge: phenotypic characterization and differentiation of human CD8+ T cells producing IL-17.. J Immunol.

[22] Zhao Y, Balato A, Fishelevich R, Chapoval A, Mann DL (2009). Th17/Tc17 infiltration and associated cytokine gene expression in elicitation phase of allergic contact dermatitis.. Br J Dermatol.

[23] Zhou L, Ivanov II, Spolski R, Min R, Shenderov K (2007). IL-6 programs T(H)-17 cell differentiation by promoting sequential engagement of the IL-21 and IL-23 pathways.. Nat Immunol.

[24] Yang XO, Pappu BP, Nurieva R, Akimzhanov A, Kang HS (2008). T helper 17 lineage differentiation is programmed by orphan nuclear receptors ROR alpha and ROR gamma.. Immunity.

[25] Ivanov II, McKenzie BS, Zhou L, Tadokoro CE, Lepelley A (2006). The orphan nuclear receptor ROR gamma t directs the differentiation program of pro-inflammatory IL-171 T helper cells.. Cell.

[26] Nicoletti F, Lamenta C, Donati S, Spada M, Ranazzi A (2008). Inhibition of human immunodeficiency virus (HIV-1) infection in human peripheral blood leucocytes-SCID reconstituted mice by rapamycin.. Clin Exp Immunol.

[27] Syrjala H, Surcel HM, Ilonen J (1991). Low CD4/CD8 T lymphocyte ratio in acute myocardial infarction.. Clin Exp Immunol.

